# The Harveian Society

**Published:** 1897-02-27

**Authors:** 


### THE HARVEIAN SOCIETY.

At the last meeting, on February 18th, Professor Herbert Spencer read a paper on "The Dangers and Diagnosis of Breech Presentation, and its Treatment by External Version towards the End of Pregnancy."

The author stated that since the days of Hippocrates the danger of birth by the breech or feet had been recognised. He gave statistics showing that from nearly one-fifth to nearly one-half of the children delivered in this manner were still-born. He discussed the causes of the high rate of mortality and the injuries (fatal and otherwise) to which the child was exposed in this method of delivery. Amongst the visceral injuries to which the child was liable he drew special attention to hæmorrhages into the liver, supra-renal capsules, lungs, testis, and spermatic cord; amongst the muscular injuries to hæmatoma of the sterno-mastoid; amongst nerve injuries to damage to the brachial plexus; and amongst bone injuries to fractures of the thigh and clavicle, the latter injury being often overlooked.

Dr. Spencer described in detail the method of diagnosing presentation of the breech by abdominal palpation, and advocated the abdominal examination of all patients at the seventh month of pregnancy, and the treatment of breech presentation by external version at seven and a half months, followed by the wearing of an abdominal belt. He mentioned six cases which he had treated in this way, and in which the children were born as vertex presentations and survived, and twenty-six cases similarly treated in the clinic of M. Pinard with one immediate infantile death, two other children subsequently succumbing, and he urged a further trial of this little operation, which was very easy, safe, and painless, and had few contra-indications.

In the discussion which followed it was pointed out by several speakers that the fatality of breech cases was not due altogether to the position of the child, but was to a considerable extent the consequence of the various abnormalities which caused the child to assume that position. It was also maintained that if all modern resources were made use of, and if everything was ready, so that no delay should take place in the delivery of the head, the fatality of breech cases need not amount to anything like what had been mentioned by Professor Spencer. The question was raised whether in some cases, in which the malposition was really an accommodation to the shortness of the cord or the position of the placenta, any attempt to rectify this position might not lead to accidental hæmorrhage.

It was also pointed out by Dr. Gow that it was not always possible to obtain an examination at the seventh month. In reply, Dr. Spencer said that he had already pointed out that there were certain contra-indications, and that in regard to the question of the cord it might sometimes occur that an abnormality of the cord might prevent an external version being successfully performed. As to the fatality, it was not correct to attribute it entirely to the fact that many cases were attended by midwives or ignorant students, or that the cases were over before the arrival of the medical man, for the statistics he had mentioned were taken from the clinic of Dr. Pinard—one of the finest in Europe. Birth by the breech must be more dangerous to the child than birth by the head. No doubt there was at present a difficulty in obtaining an examination at or about the seventh month, but that was to some extent the result of such an examination not being systematically asked for. Women were, he thought, quite willing to submit to anything which they believed was for their own safety, and this was to a large extent a matter of custom. In Germany such examinations were customary, and there was no difficulty; he had found that ladies who had been abroad, on making arrangements for their confinement would now ask, "When would you like to examine me?" and he was sure that for many reasons the advantages of obtaining such an examination were very great.

